# Mechanical Processing of *Hermetia illucens* Larvae and *Bombyx mori* Pupae Produces Oils with Antimicrobial Activity

**DOI:** 10.3390/ani11030783

**Published:** 2021-03-11

**Authors:** Alessio Saviane, Luca Tassoni, Daniele Naviglio, Daniela Lupi, Sara Savoldelli, Giulia Bianchi, Giovanna Cortellino, Paolo Bondioli, Liliana Folegatti, Morena Casartelli, Viviana Teresa Orlandi, Gianluca Tettamanti, Silvia Cappellozza

**Affiliations:** 1 Consiglio per la Ricerca in Agricoltura e l’Analisi dell’Economia Agraria, Centro di Ricerca Agricoltura e Ambiente (CREA-AA), 35143 Padova, Italy; alessio.saviane@crea.gov.it; 2 Istituto Zooprofilattico Sperimentale delle Venezie, Legnaro, 35020 Padova, Italy; ltassoni@izsvenezie.it; 3 Dipartimento di Scienze Chimiche, Università di Napoli “Federico II”, 80126 Napoli, Italy; naviglio@unina.it; 4 Dipartimento Scienze per gli Alimenti, la Nutrizione e l’Ambiente, Università degli Studi di Milano, 20133 Milano, Italy; daniela.lupi@unimi.it (D.L.); sara.savoldelli@unimi.it (S.S.); 5 Consiglio per la Ricerca in Agricoltura e l’Analisi dell’Economia Agraria, Centro di Ricerca Ingegneria e Trasformazioni Agroalimentari (CREA-IT), 20133 Milano, Italy; giulia.bianchi@crea.gov.it (G.B.); giovanna.cortellino@crea.gov.it (G.C.); 6 Freelance Expert, 20133 Milano, Italy; paolo.bondioli1956@gmail.com; 7 Innovhub, Laboratorio Sostanze Grasse, Derivati e Tecnologie Olearie, 20133 Milano, Italy; liliana.folegatti@mi.camcom.it; 8 Dipartimento di Bioscienze, Università degli Studi di Milano, 20133 Milano, Italy; morena.casartelli@unimi.it; 9 Dipartimento di Biotecnologie e Scienze della Vita, Università degli Studi dell’Insubria, 21100 Varese, Italy; viviana.orlandi@uninsubria.it (V.T.O.); gianluca.tettamanti@uninsubria.it (G.T.)

**Keywords:** black soldier fly, silkworm, fat, oil extraction method, antimicrobials, agar diffusion assay, Gram-positive, Gram-negative

## Abstract

**Simple Summary:**

Insect rearing and processing are widely studied to provide solutions to the increasing demand for proteins caused by a growing human population. It has been demonstrated that insect meal can be introduced into the feed ratio of livestock and its exploitation can limit the environmental impact of animal husbandry. When their ecological footprint is analyzed, insects are considered “biorefineries” able to generate multiple economic outputs for the food industry. Fat and antimicrobial peptides contained in the insect oil can represent important resources to treat animal diseases, e.g., allowing to reduce antibiotic overuse. In this paper we focused on two insects exploitable both for protein and oil production: the black soldier fly larvae (BSFL) and the domestic silkworm. As the diet regimen remarkably affects the fat composition of the insects, both BSFL and silkworm larvae were reared on plant-based diets. Silkworms were fed mulberry leaves, while BSFL were reared on a diet composed of leftover vegetables and fruit, in the perspective of their bioconversion and valorization. The best technique to maximize the oil extraction yield was defined for BSFL and applied to the silkworm pupae. An antimicrobial activity of the oil against some bacterial species was demonstrated in both insects and compared.

**Abstract:**

The aim of this work was to develop processing methods that safeguard the quality and antimicrobial properties of *H. illucens* and *B. mori* oils. We adopted a vegetable diet for both insects: leftover vegetables and fruit for *H. illucens* and mulberry leaves for *B. mori*. First, alternative techniques to obtain a good oil extraction yield from the dried biomass of *H. illucens* larvae were tested. Traditional pressing resulted to be the best system to maximize the oil yield and it was successfully applied to *B. mori* pupae. Oil quality resulted comparable to that obtained with other extraction methods described in the literature. In the case of *B. mori* pupae, different treatments and preservation periods were investigated to evaluate their influence on the oil composition and quality. Interestingly, agar diffusion assays demonstrated the sensitivity of Gram-positive *Bacillus subtilis* and *Staphylococcus aureus* to *H. illucens* and *B. mori* derived oils, whereas the growth of Gram-negative *Pseudomonas aeruginosa* and *Escherichia coli* was not affected. This study confirms that fat and other active compounds of the oil extracted by hot pressing could represent effective antimicrobials against bacteria, a relevant result if we consider that they are by-products of the protein extraction process in the feed industry.

## 1. Introduction

Insects are considered optimal bio-converters of non-food biomass derived by agricultural processes. In particular, *Hermetia illucens* (black soldier fly) larvae (BSFL) can feed on several different organic materials [1], while *Bombyx mori* (domestic silkworm) pupae (SP) are by-products of the agro-industrial supply chain dedicated to silk production. Their multipurpose, low-cost, and environmentally sustainable rearing make them attractive for the feed industry as a source of proteins, fat, and bioactive compounds. Insect oil is rich in antimicrobial ingredients, which might be very useful to protect human and animal health from pathogenic bacteria. The antibacterial properties of BSFL fat have already been investigated [2,3], while the use of SP in traditional Eastern Asian medicine dates back to ancient times, although, as far as we know, the first attempts to systematically study the antimicrobial compounds of pupal oil are very recent [4].

In the last decades, human behaviour inadvertently favored the selection of multiresistant bacterial strains and helped them to spread. In fact, antimicrobials have often been used inappropriately to control human diseases (antibiotic prescription for viral infections, interruption of the antibiotic therapy before bacteria eradication, use of sub-standard or counterfeit antibiotics) [5] or misused for the treatment and prevention of diseases in livestock, aquaculture, and even for crop production [6]. Therefore, presently there is an urgent need to identify new antimicrobials, especially of natural origin, and for this reason, it is advisable to approach “the reservoir of untapped natural products, which is likely the next antibiotic gold mine” [7]. Insects can represent a good source of new antimicrobial compounds, especially with the aim of using them for animal feeding, for which we currently have blunt weapons only [8].

Based on this global scenario, we began to study the effects of insect oil on microbial pathogens. The insect processing industry, still at an early stage of development, largely borrowed its processes from other production supply chains, and needs to make significant improvements in order to obtain insect oil of good quality in an economically-viable quantity [9]. Consequently, the general aim of the present paper was finding an efficient and simple method to extract high quality oil from insects and evaluating its antimicrobial activity. For this purpose, we selected two insect species which are widely and easily reared, far from a taxonomic point of view (*H. illucens* is a fly, belonging to the Diptera order, while *B. mori* is a moth, belonging to Lepidoptera), and therefore representative of different criticalities. The first specific objective of our research was to develop extraction methods that guarantee the maintenance of the characteristics of insect fat as intact as possible and comparable to the quality already documented in the literature. Furthermore, in the case of SP, a further specific aim was to evaluate how different preservation conditions of SP or their recovery following silk reeling can influence the quantity and quality of the extracted fat. Our last specific goal was to preliminarily assay the antimicrobial activity of the oils derived from both insects to evaluate their efficacy against pathogens and to assess whether the processing technique was respectful of the active constituents of the oils.

## 2. Materials and Methods

### 2.1. Insect Rearing

*H. illucens* larvae were mass-reared as previously reported [10]. Briefly, three replicates of ≈ 5000 eggs (obtained from the colony collected from a wild population in Lombardy–Italy in 2015 and then maintained under lab conditions) were directly put on a diet composed of a mix of fruit and vegetables (leftovers of the vegetable and fruit market) cut into small pieces of about 5 mm, in plastic containers (dimension: 40 cm × 60 cm × 5 cm; volume: 12 L) where a perforated bottom assured drainage of excessive humidity. A series of preliminary tests was carried out to establish the optimum quantity of feed with this sample size, that resulted to be 120 mg VDM larva^−1^ day^−1^ (wet weight). At the end of the larval cycle, BSFL were collected, freezed, and then dried.

*B. mori* polyhybrid larvae (121 × 125) (74 × 118) were reared from hatching to the beginning of the third instar at CREA—Sericulture laboratory of Padua and then distributed to farmers for rearing of the last three instars. Larval rearing on mulberry leaves was performed according to the routine procedure [11] until spinning. After a week from larval spinning, fresh cocoons were removed from plastic mountages, deflossed, sorted, and dried at CREA—Sericulture laboratory of Padua.

### 2.2. Insect Drying

*H. illucens* larvae were dried at 70 °C in a cocoon dryer [10]. *B. mori* pupae were dried in their cocoons, according to the procedure usually adopted in the silk industry, at 70 °C until cocoons reached 42–44% of their fresh weight.

### 2.3. B. mori Cocoons Treatments

While *H. illucens* larvae were dried and then immediately subjected to a defatting treatment (see below), *B. mori* required the exploration of different treatments preceding defatting to replicate the real conditions of the silk production process of SP (long-term preservation and reeling). Four experimental theses were compared: (A) SP produced 4–6 weeks before the analysis (freshly produced pupae) were dried in their cocoons after a week from cocoon production; the dried cocoons were immediately cut, pupae extracted and maintained at 5 °C in plastic bags under vacuum in the dark; (B) SP produced in the same year of the analysis (3–4 months before) were dried in their cocoons after a week from cocoon formation; the dried cocoons were preserved in plastic bags at room temperature, cut a week before the analysis and the pupae extracted and maintained at 5 °C in plastic bags under vacuum in the dark; (C) SP produced one year and three months before the analysis, were dried in their cocoons after a week from cocoon formation; dried cocoons were preserved in plastic bags at room temperature, cut a week before the analysis and the pupae extracted and maintained at 5 °C in plastic bags under vacuum in the dark; (D) SP produced in the same year of the analysis (3–4 months before) were dried in their cocoons after a week from cocoon formation; then, dried cocoons underwent the reeling process until the silk was completely unraveled. As the reeling process involves cocoon cooking at high temperature and pressure in an autoclave and subsequent dipping in hot water in the reeling basins, the reeling leftovers were dried again at 70 °C and then preserved in plastic bags at room temperature until pressing; one week before the oil extraction process, the residual silk was manually removed and pupae were maintained in plastic bags, under vacuum at 5 °C, in the dark.

### 2.4. Extraction Tests

(1) Solvent extraction: this extraction was carried out to quantify the exact amount of oil contained in the BSFL; however, chemical extraction was not considered as a possible option from the perspective of a circular economy. The larvae were pressed and three meal replicates of 10 g each were extracted with solvents in 100 mL of ether, at room temperature, for 30 min, under continuous agitation using a magnetic stirrer. Then, the mixture BSFL meal/solvent was transferred into a separating funnel with 2 mL of a 20% NaCl solution; after the elimination of the aqueous phase, the lipophilic phase was centrifuged for 20 min at 15,000 rpm at 4 °C. The meal residue was re-extracted with 100 mL of ether for 15 min. The two ether extracts were pooled together, evaporated to dryness at 30 °C using a rotating evaporator, and then weighed to determine the amount of extracted lipids.
(2) Aqueous extraction:
  (a) Meal aqueous extraction: BSFL were ground, then 100 g of the meal were added to 500 mL of cold water in a beaker on a heating plate under agitation by magnetic stirrer. Thermoregulation was set at 50 ± 2 °C during the extraction phase (3 h). Then, the mixture meal/water was poured into an Imhoff cone, which was maintained at a 2 °C for 48 h. Finally, the separation state of the lipid phase at the surface and the meal sedimentation were checked.
  (b) Whole larva aqueous extraction: 50 g of dried larvae and 500 mL of water pre-heated at (i) 70 °C or (ii) 100 °C were put into flasks with taps on heating plates and agitated with magnetic stirrers. Thermoregulation was set at (i) 70 ± 2 or (ii) 100 ± 2 °C during the extraction phase (1 h). Then, the larvae were drained and the extraction water from the two samples at different temperatures was poured into two separate Imhoff cones, which were maintained at 2 °C overnight, to evaluate the quantity of extracted lipids that emerged to the water surface.
(3) Naviglio extraction: the extracts were obtained by using a rapid solid–liquid dynamic extraction (RSLDE) through a Naviglio Extractor (NE) (AtlasFiltri Engineering, Padua, Italy). This equipment allows a rapid solid–liquid extraction, maintaining the liquid in contact with the solid in programmable pressurization–depressurization cycles. The functioning of Naviglio Extractor [12,13] is based on a suction effect, generated by the compression of an extracting solvent on solids at a pressure of about 6-8 bar for a determinate time, and followed by an immediate decompression at a pressure of about 1 bar, i.e., atmospheric pressure. The rapid release of the extracted liquid from the solid matrix, as a consequence of the pressure gradient, mechanically transports the extractable compounds contained in the solid matrix into a solution.
  (a) Test 1 (maceration at 80 °C and NE extraction for 1 h): 50 g of dried larvae were placed in 600 mL of distilled water at 80 °C for 5 min. After the blanching process, the material was transferred to the 500 cc NE chamber for 1 h (static phase: 1 min; dynamic phase: 2 min).
  (b) Test 2 (NE extraction with water/ethanol 50:50 (*v*/*v*) for 24 h): 50 g of dried larvae were extracted with 600 mL of the hydroalcoholic solution using the 500 cc NE chamber for 24 h (static phase: 1 min; dynamic phase: 2 min).
  (c) Test 3 (NE Extraction with 96% ethanol (*v*/*v*) for 24 h): 50 g of dried larvae were extracted with 600 mL of 96% (*v*/*v*) ethyl alcohol by means of the 500 cc NE chamber for 24 h (static phase: 1 min; dynamic phase: 2 min).

In all the tests the larvae were dried in an oven at 70 °C for 12 h. The weight of the dry material was recorded. The extracted lipid component was determined through liquid-liquid extraction by mixing the resulting liquid with hexane.

### 2.5. Crushing of BSFL

The BSFL dried biomass was pressed for obtaining oil and partially defatted meal. A continuous screw press mod. PC 25 (MIG, Fornovo San Giovanni, BG, Italy) was used. The equipment was constituted of a loading hopper, which was filled with the dried biomass, and a conveyor belt for material transfer into the continuous heater. The biomass arrived into the heater installed over the press where the temperature was kept at 80 °C by means of indirect heating. The pre-heated material was transferred into the press by a vibrating device. The dried BSFL biomass was pressed using a screw-press rotating at approximately 20 revolutions per minute (rpm). To remove all solid impurities the obtained oily material was filtered while it was still hot.

### 2.6. Crushing of SP

The SP dried biomass was pressed for obtaining oil and partially defatted meal. A laboratory screw press mod. Komet (IMG Monfort, Moenchengladbach, Germany) was used for the test. The equipment was constituted of a loading hopper, which was filled with dried biomass and then transferred by simple drop to the press, whose head was heated at 100–120 °C. The press rotated at approximately 4 rpm. The nozzle diameter was 6 mm. To remove all solid impurities the obtained oily material was filtered while it was still hot (80 °C). Thesis A (freshly produced pupae, see above) was used to calculate the oil recovery.

### 2.7. Chemical Analyses

On insect biomass (BSFL and SP):

- Humidity and volatile matter: these properties were evaluated by means of weight loss in a thermostatic oven set at 103 °C [14].
- Total fat content: the fatty substances were extracted from a test sample with classic Soxhlet extraction using hexane, according to [15].

On oils:

- Free fatty acids were determined by means of volumetric titration using phenolphthalein as an indicator using standard method [16].
- Peroxide value was determined by means of volumetric titration based on the liberation of iodine from potassium iodide in presence of hydroperoxides. A starch aqueous solution was used as an indicator according to [17].
- Fatty acid composition was determined according to [18]. The analyses were carried out by a gas-chromatograph FOCUS (Thermoquest Instrument, Rodano, Italy) equipped with a flame ionization detector (GC-FID), using a capillary column (CP-Sil 88−l = 100 m, 0.32 mm i.d., film thickness 0.25 μm; Supelco, Bellefonte, PA, USA) after derivatization of fatty acids into the corresponding methyl esters, under the following experimental conditions: carrier gas He at a flow rate of 1.5 mL/min; split injection system with a splitting ratio 1:40; injector and detector temperatures set at 250 and 260 °C respectively; using the following program: 90–240 °C at 7 °C/min; injected quantity 1 μL.

### 2.8. Antimicrobial Assays

- Bacterial strains and growth conditions: *Pseudomonas aeruginosa* PAO1, *Escherichia coli* C1a, as Gram-negative, and *Staphylococcus aureus* ATCC 6538P and *Bacillus subtilis* ATCC 6633, as Gram-positive, were selected as model microorganisms to test the antimicrobial activity of *H. illucens* and *B. mori* oil. The strains were grown overnight in Luria-Bertani (LB) medium at 37 °C under 200 rpm shaking.
- Agar diffusion test: the antimicrobial activity of *H. illucens* and *B. mori* oil was tested by agar diffusion assay. Briefly, overnight LB bacterial cultures were inoculated on LB agar plates and let dry. A volume of 10 μL of oil or oil dissolved in solvents was loaded onto the inoculated plates. The oil samples were diluted 5-fold or 2-fold in dimethylsulfoxide (DMSO), *N*,*N*-dimethylformamide (DMF) and benzyl benzoate (BB). The activity of the solvents was checked as control. The plates were incubated at 37 °C for 24 h and the antibacterial effect was evaluated by measuring the growth inhibition halo. All experiments were performed in triplicate.

### 2.9. Statistical Analysis

Chemical analyses were carried out in triplicate (BSFL) and standard deviation was calculated. For SP all tests were carried out in duplicate using standardized methods [14,15,16,17,18]. The average value of two independent determinations is reported, after repeatability check, ±expanded uncertainty of measurement (95%).

The diffusion tests were performed at least three times with different cultures. Data were analyzed by means of Mann-Whitney non-parametric test (Statistica 8, StatSoft, Tulsa, OK, USA). Significant effects of treatments were estimated (*p* < 0.05).

## 3. Results

The different extraction tests performed on BSFL were compared to identify the best procedure in terms of oil yield. The lipid content of larvae chemically extracted with ether was 29.55 ± 1.21% of the dry weight (DW).

### 3.1. BSFL Aqueous Extraction

#### 3.1.1. Meal Aqueous Extraction

After 48 h at 2° C, the meal remained in suspension, without settling on the bottom of the cone; furthermore, the extracted lipid quantity appeared to be very scarce as no solid lipid phase was present at the surface (Supplementary Materials—Figure S1B). Lipid extraction from the meal with water at 50 °C was not successful for two reasons: (1) the meal, after rehydration with water, did not settle down (Supplementary Materials—Figure S1A), therefore its separation from water containing the lipids was not feasible; (2) the “mild” temperature conditions did not allow a good lipid recovery.

#### 3.1.2. Whole Larvae Aqueous Extraction

From the two tests at different temperature, it was evident that the amount of extracted lipids raised as long as the water temperature increased from 70 to 100 °C (Supplementary Materials—Figures S2 and S3); on the other hand, the use of whole larvae permitted to easily separate lipids from water (Supplementary Materials—Figures S2B and S3C).

Nevertheless, the amount of the solidified lipid phase, which could be isolated from water and represented the total extracted lipids, was much lower (about 4 g/100 g DM at 70 °C, 6.55 g/100 g DM at 100 °C) than the quantity extracted with the solvent (about 29.55 g/100 g DM) (Table 1).

### 3.2. Tests with Naviglio Extractor

In the tests performed with the Naviglio extractor (Table 2) the quantity of extracted lipids was very low, even compared to water extraction only (Table 1). In addition, not only lipids were extracted, but other components too, which should imply further purification.

### 3.3. BSFL Pressing Process Efficiency

The dried biomass (72 kg were crushed at once) (Supplementary Material—Figure S4a) was analyzed before mass pressing and defatting, to evaluate total water and oil content, which resulted in 1.90% moisture and 35.62% crude ether extract. This value was slightly different compared to that previously indicated (30.61 g/100g DM) (Table 1) because of the seasonal variation of vegetable and fruit market leftovers. Thus, the total biomass was processed obtaining 18.8 kg of oily material (87.0% oil, 1.7% humidity, 11.3% impurity), and 53.2 kg (73.9% of the initial dry larvae amount) of the partially defatted meal (humidity: 1.96%, crude ether extract: 17.45%). Therefore, the calculated oil recovery was 64%.

### 3.4. SP Pressing Process Efficiency

The dried biomass (thesis A) (1379 g were crushed at once) (Supplementary Material—Figure S4b) was analyzed before laboratory pressing and defatting, to evaluate total water and oil content, which resulted in 5.89% moisture and 30.01% crude ether extract. Thus, the total biomass was processed obtaining 387 g of oily material (83.9% oil, 6.9% humidity, 9.2% impurity), and 992 g (71.9% of the initial dry larvae amount) of partially defatted meal (humidity: 5.49%, crude ether extract: 9.01%). Therefore, an oil recovery of 78.4% was calculated.

### 3.5. Chemical Analyses of BSFL Non-Defatted Meal and Oil

The results of the solvent extraction of the non-defatted meal and of the oil obtained by mechanical pressing, the ratio of saturated fatty acids (SFA) vs. unsaturated fatty acids (UFA) and that of ω6/ω3 were investigated (Supplementary Material—Table S1).

The extraction method can give different results in terms of relative proportions of fatty acids in the oil, although qualitative differences can be considered very low (Supplementary Material—Table S1).

### 3.6. Chemical Analyses of B. mori Meal and Oil

In the case of *B. mori* pupae the experimental material was divided into four theses (A, B, C, D) (Table 3), as reported in Materials and Methods.

Lauric acid was the prevalent fatty acid (Supplementary Material—Table S1) in BSFL oil, given the vegetable substrate used in this experiment; in the SP, linolenic acid (ω-3) represented the prevalent part at par with oleic acid, while a discrete amount of linoleic acid (ω-6) was also present (Table 3). Linoleic acid (ω-6) was present in similar quantities in the two insect oils. The comparison of the four experimental theses demonstrated that the oils from *B. mori* pupae had very similar compositions in terms of fatty acids with very low differences that could also be due to seasonal variations of mulberry leaves composition between spring and summer or among different years. Therefore, the silkworm oils were also evaluated in terms of their peroxide number and oil acidity, as defined in Materials and Methods (Table 4).

### 3.7. Antimicrobial Assays

**Figure 1:**
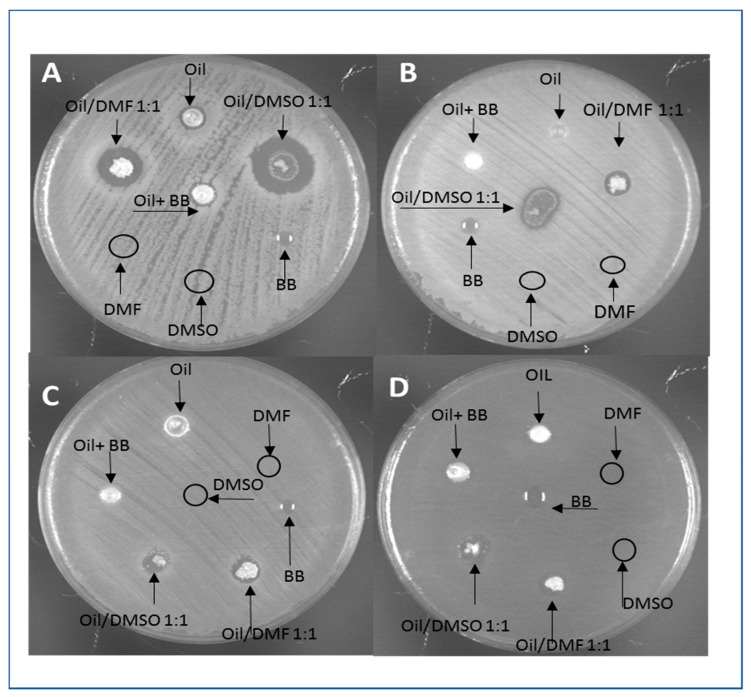
Representative images of Agar diffusion tests. Samples of *H. illucens* oil or oil mixed 1:1 with dimethylsulfoxide (DMSO), *N*,*N* Dimethylformamide (DMF) and Benzyl benzoate (BB) were loaded on LB agar plates inoculated with *B. subtilis* (**A**), *S. aureus* (**B**) *E. coli* (**C**), and *P. aeruginosa* (**D**). Upon 24-h growth, inhibition halos were observed and measured.

The potential antimicrobial activity of *H. illucens* and *B. mori* oils was assessed through agar diffusion tests. *B. subtilis* and *S. aureus* were chosen as representative strains of Gram-positive bacteria, while *E. coli* and *P. aeruginosa* as Gram-negative ones. The agar diffusion test did not highlight any significant antimicrobial activity of *H. illucens* oil against the chosen microbial testers. In fact, Gram-negative species were completely tolerant to this treatment, while Gram-positive showed a very low sensitivity (Figure 1).

The five-fold dilution of *H. illucens* oil in DMSO, DMF or benzyl benzoate did not influence the antimicrobial activity (data not shown). It was necessary to mix *H. illucens* oil with solvents at a higher ratio (1:1) to observe growth inhibition halos of Gram-positive bacteria (Figure 1A,B). *E. coli* and *P. aeruginosa* were insensitive to all treatments (Figure 1C,D).

As benzyl benzoate was toxic to all tested microorganisms, the effect of oil mixed with BB on bacteria was not considered. On the other hand, as DMSO and DMF did not show any intrinsic toxicity, the antimicrobial activity induced by *H. illucens* oil mixed with DMSO or DMF was considered ascribable to oil component/s. Indeed, mixing oil with DMSO determined a growth inhibition halo greater than oil-DMF in both Gram-positive species, *B. subtilis* and *S. aureus*. The former was more sensitive to both treatments than the latter (Table 5).

**Figure 2:**
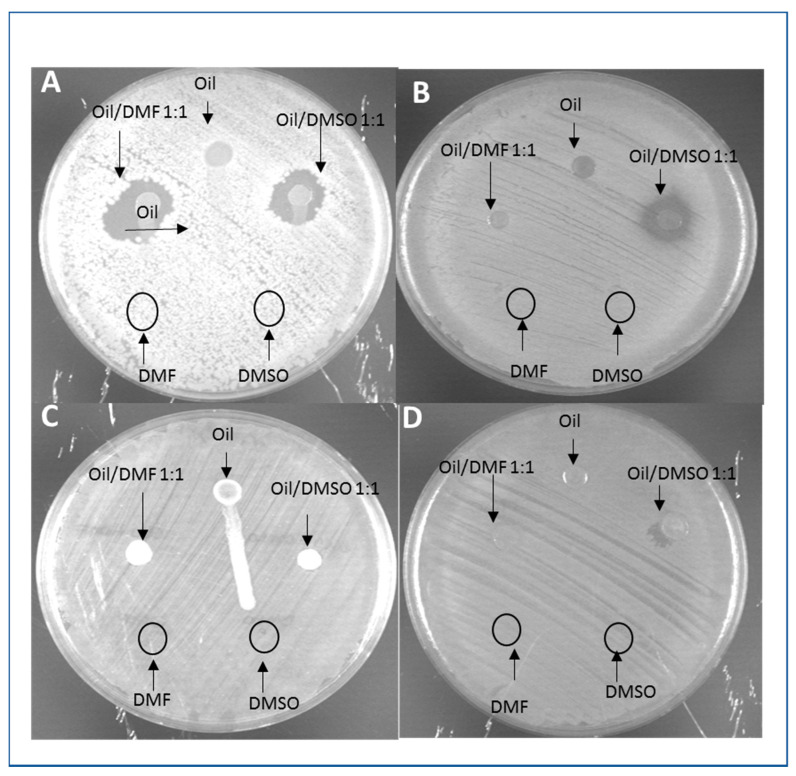
Representative images of agar diffusion tests. Samples of *B. mori* oil or *B. mori* oil mixed 1:1 with dimethylsulfoxide (DMSO) or *N,N*-dimethylformamide (DMF) were loaded on LB agar plates inoculated with *B. subtilis* (**A**), *S. aureus* (**B**) *E. coli* (**C**), and *P. aeruginosa* (**D**). Upon 24-h growth, inhibition halos were observed and measured.

*B. mori* oil was tested alone and mixed with DMSO or DMF (1:1). The treatment of oil with solvents permitted to observe a selective antimicrobial activity. In fact, while Gram-positive bacteria were sensitive to the oil (Figure 2A,B), *E. coli* and *P. aeruginosa* were not sensitive (Figure 2C,D). Furthermore, DMSO was the best solvent to extract potential antimicrobials (Table 5).

## 4. Discussion

### 4.1. Generalities

The choice of extracting oil from insects, instead of using the whole insect as feed for farmed animals, is due to the consideration that insects can only partially replace protein and fat usually introduced into the feed ratios of domestic animals. These compounds of the diet mostly derive from soybean and fishmeal; moreover, livestock and fish species have different requirements. Therefore, the chance of separately manipulating insect protein, different amino acids, and fat, and to add them in appropriate quantities to the feedstuff, might represent a great advantage for the feed industry. For these reasons we decided to investigate the oil extraction technique from insects. Since insect biomass preservation and oil extraction can affect the oil yield, and its antimicrobial activities, we tested different extraction methods for *H. illucens* and applied the best one to *B. mori* pupae; then, for SP, we tested different treatments of the insect biomass preceding oil extraction, to see their impact on fat quality. The present study demonstrated that larvae/pupae hot pressing maximized the oil yield both in BSFL and SP.

It is well-known that the insect species, the rearing substrate, and the extraction method have a great influence on the quantity and quality of the oil yield. The two insects selected for our study, although taxonomically different, have some similarities that facilitate a comparison: (1) they can be used in circular economy settings giving place to considerable economic outputs; (2) they can be reared on vegetable substrates, therefore their oil profile can be mostly composed of unsaturated fatty acids with very interesting properties, as shown in the present work (Supplementary Material—Tables S1 and S3); (3) their rearing substrate can be modulated and enriched; in fact, different feeds have been tested for BSFL [10,19,20] and several artificial diets, with different compositions, were studied for the silkworm [21,22,23]; furthermore, different diet regimens affect the fatty acid composition of the two insects [24,25]; and (4) it has already been demonstrated they are rich of antimicrobial peptides and other compounds with pharmacological action [2,3,26].

### 4.2. Comparison of BSFL and SP Derived Oil Obtained in this Study with Literature Data

A comparison of the composition of BSFL and SP oil performed in this and other studies (Supplementary Material—Figure S5) [19,27,28,29,30,31,32] demonstrated that BSFL oil contains less polyunsaturated fatty acids (PUFA) and monounsaturated fatty acids (MUFA) than SP oil and more saturated fatty acids (SFA). To compare our data on BSFL with those reported in the literature, we choose studies in which larvae were reared on vegetable substrates. It is possible to observe that the relationship among the three groups of fatty acids has slight variations according to the insect feed, as it is quite characteristic of the species (Supplementary Material—Figure S5). In fact, the fatty acid profile of BSFL oil is similar to that already reported for two different diets (palm kernel meal and 80% industrial waste mix/20% organic waste) [33]. Therefore, in accordance with [33], the BSFL diet impacts on the relative nutrient composition (especially the quantity of lipids vs proteins), but does not substantially affect fatty acid composition, with lauric acid being the dominant fatty acid of BSFL oil. The same can be observed for SP (Supplementary Material—Figure S5). Furthermore, it is noteworthy that fatty acid composition is slightly different according to the extraction method, and chemical or mechanical extraction can affect the quantity of saturated vs. unsaturated fatty acids and decrease ω-6/ω-3 ratio (Supplementary Material Table S1 and Figure S5).

### 4.3. Extraction Method Efficiency in Relation to Fat Distribution in the Insect Body

Different studies focused on lipid extraction from insects using simply water as it represents a more eco-sustainable approach than chemical solvents like hexane; however, it was demonstrated that aqueous extraction affects the oil yield in other insects to a considerable extent [34,35]. This loss occurs because lipids are retained by insoluble-solids or the oil-in-water emulsion [34]. In complete agreement with these previous findings, we obtained very scarce results when we applied an extraction in water even at high temperature (100 °C) (Table 1) to avoid chemical residuals in oils. For this reason, we resorted to the Naviglio^®^ extractor that has already been successfully applied to vegetal substrates for solid/liquid extraction. This new method differs from other extractive techniques based on diffusion and osmosis and depending on temperature, therefore it is also respectful of thermolabile substances. The reported test has a novel character because this extraction process has not been evaluated on insects so far, but unfortunately gave no valuable results on BSFL, even using mild solvents (ethanol or citric acid) instead of simple water (Table 2). This technical failure could be due to the anatomy of the fat body, the organ which is responsible for metabolism and accumulation of fat in insects. In fact, it is a relatively large organ distributed throughout the insect body, preferentially underneath the integument and surrounding the gut and reproductive organs [36]. The fat body is structurally heterogeneous and exhibits regional differentiation that can be distinguished morphologically [37]. Furthermore, the role of the fat body changes according to the insect life stage: it is very different in BSFL, which represent the insect feeding stage, or in SP, which represent the intermediate step to the reproductive stage. In SP the fat body is mainly perivisceral; this tissue is far more abundant in female pupae, in which fat cells are progressively destroyed to obtain egg maturation [38]. Recently, other methods were proposed for fat extraction, as mechanical and enzyme-assisted fractionation process, keeping into account the particular distribution of fat in BSFL [39]. However, in the present study we resorted to mechanical crushing of the dried biomass, which is a quite simple method, already tested for plant seeds, and which requires slight modification of the already existent technologies. The difference in efficiency of oil recovery between the two insects (64% for *H. illucens* versus 78.4% for *B. mori*) can be due to the different scale of oil extraction (mass pressing for *H. illucens* larvae, laboratory pressing for *B. mori* pupae), but mostly to the different location of the prevalent part of the fat body in the two insects, processed at two developmental stages. Few reports on the extraction yield of oil obtained from insect mechanical pressing, particularly from BSFL, are available in the literature and mostly refer to freeze-dried or blanched insects. However, the percentage of oil recovered (64%) in this study by mass oil extraction, was satisfying if compared to the 40% oil recovery obtained by Surrendra et al. [40].

### 4.4. Impact of SP Preservation on Oil Quality and Extraction Efficiency

For *B. mori*, in which oil is a by-product of the silk industry, we compared different theses to evaluate whether it can be used as a feed component or antimicrobial substance. The number of peroxides in unsaturated fats and oils is a measure of rancidity caused by the action of light, humidity, air, high temperature, and endogenous enzymes. It is evident that the peroxide number was acceptable for all the samples, being < 15 (the limit value for the extra virgin olive oil is 20 mEq O_2_/kg) in all the samples and < 7 in three out of four samples. According to [41] the number of peroxide in the insect oil should be between 10 and 20 mEq O_2_/kg; the highest value shown by the D thesis might be due to multiple thermal treatments necessary for silk thread unravelling, consisting of cocoon steaming and dipping in hot water during cooking and reeling. The second highest value of peroxides was recorded in the B thesis and can be related to a high water content of dried pupae in this sample (Table 5). In the same way, a high water content found in the C thesis might be responsible for the excessive acidity of this sample, preserved for more than a year. Therefore, it is important to outline that the external silk shells, in which the pupae were preserved until the analysis, cannot protect against re-hydration under room conditions, and the preservation should be performed accordingly. In fact, the moisture value suggested for good preservation is <5%. On the other hand, oil acidity is a measure of the free fatty acids (FFA) present in the fat or oil. An increment in the amount of FFA in a sample of oil or fat indicates hydrolysis of triglycerides. Such reaction occurs because of the action of lipases and it is an indicator of inadequate processing and storage conditions (i.e., high temperature and relative humidity, tissue damage) [42]. It is also important to notice that the deterioration of the oil quality also causes a decrease in the extractable oil quantity (D < C < B < A) (Table 4). This study demonstrated that when the pupae are dried immediately after reeling, they might represent an acceptable material for oil extraction, although the best result is obtained with pupae dried and extracted immediately after the rearing season. On the other hand, long preservation at room temperature and humidity resulted in an excessive increase of acidity, probably caused by rehydration of dried pupae. In general, our study is one of the few reports about acidity of insect oils. For *H. illucens*, it was recently stated that crushing gives better results than bleaching and freezing, with regard to this parameter, so that the acidity value obtained with this kind of extraction was 2.5% free fatty acids [43].

### 4.5. Antimicrobial Activity of BSFL and SP Oils: Possible Role of Fatty Acids

Our preliminary microbiological investigations showed an antimicrobial activity of *H. illucens* and *B. mori* oils with a very similar efficacy. It is noteworthy that a simple treatment of both oils with polar solvents such DMSO and DMF was enough to extract component/s that inhibit bacterial growth of the chosen Gram-positive model strains. *S. aureus,* which is an important etiological agent of nosocomial infections and *B. subtilis,* which is a spore-forming microorganism, are susceptible to both oils. Considered as a whole, the use of DMSO seems better than DMF to enhance the antimicrobial activity of both oils (Table 5). *H. illucens* oil dissolved in DMSO showed a significantly higher (*p* < 0.05) antimicrobial activity than in DMF against *B. subtilis*. *B. mori* oil treated with DMSO and used against *S. aureus*, induced an inhibition higher than that observed with DMF (*p* < 0.05). Furthermore, *B. subtilis* was more sensitive than *S. aureus* to *H. illucens* oil dissolved in DMSO (*p* < 0.05). On the basis of the above-described results, the hydrophobic nature of the unidentified oil antimicrobial/s can be inferred and, among these, fatty acids are the most likely candidates. It is known that antimicrobial potential of fatty acids depends on hydrocarbon chain length, unsaturation, and presence of functional groups [44].

Both *H. illucens and B. mori* oils were highly selective for Gram-positive microorganisms. It has been reported that Gram-positive bacteria are sensitive to fatty acids, while very few Gram-negative species are sensitive to these molecules and if so, to a lower extent. This is in accordance with the hypothesis that fatty acids are probably involved in the mechanism of action of the oils tested in this study. In fact, Gram-positive bacteria have a cell wall constituted by a single thick peptidoglycan layer only, while Gram-negative bacteria, although characterized by a thin peptidoglycan layer, present an outer membrane layer composed of lipoproteins, lipopolysaccharides, and phospholipids [45]. This layer of Gram-negative bacteria prevents the entry of intermediate and long-chain fatty acids and their subsequent toxic action [44]. The antibacterial activity of long-chain unsaturated fatty acids is stronger for oleic, linoleic, linolenic acid, while long-chain saturated fatty acids, including palmitic and stearic acid, are less active [46]. *B. mori* oil shows a prevalence of long-chain unsaturated fatty acids (Table 3), which are mostly derived from the vegetal source used as a rearing substrate for the larvae; *H. illucens* oil also contains these fatty acids in a significant quantity, especially oleic acid (Table 3). In addition, *H. illucens* oil is characterized by a prevalent amount of lauric acid (Table 3), which is a medium-chain fatty acid and has also a broad spectrum of activity against different bacteria in vitro [47,48]. The quantity and quality of fatty acids, especially in *H. illucens* larvae, can differ with the progression of the larval instars and the use of different growing substrates [24]; however, BSFL oil always contains a good quantity of lauric acid. Silkworm oil presents a higher amount of PUFA if compared to BSFL oil (Supplementary Material—Figure S5). PUFA and free fatty acids have also been identified as antimicrobials with a broad spectrum of activity, in addition, not affected by classical resistance mechanisms [49,50,51].

### 4.6. Antimicrobial Activity of BSFL and SP Oils: Possible Role of AMP

Insects oils are rich in other putative antimicrobials such as antimicrobial peptides (AMP), polymers, and chemical complexes that could act alone or synergically with fatty acids [34,35,52,53]. A recent “in silico” transcriptomic analysis of *H. illucens* brought to the identification of 57 peptides with putative antimicrobial activity and belonging to cecropin and defensin families [54]; on the other hand, six families of AMP have been identified in *B. mori*, namely cecropin, defensin, moricin, gloverin, attacin, and lebocin [55].

The attribution of an antimicrobial role to AMP in the oil, once the fat has been extracted from the insect, is still premature because of the lack of knowledge in this field. Their action, which could not be studied in the present research, should also be evaluated in further experiments on *H. illucens* and *B. mori* oils.

## 5. Conclusions

The study presented in this paper focused on finding an efficient and simple method to extract oil from two insect species at different developmental stages, BSFL and SP, by maintaining the characteristics of their fat as intact as possible. Processing techniques were evaluated in the context of a circular economy scenario, with the aim of producing oil and protein from BSFL, and oil and silk from SP. Mechanical pressing of dried insects appeared to be a good compromise in terms of oil recovery and it ensured a good quality of oil. Furthermore, it did not imply the design of new technologies, and the methodology was adapted from the industry of oil extraction from plant seeds.

In general, from these first and preliminary tests, an antimicrobial activity of the BSFL and silkworm oil was inferred; this was probably due to unsaturated long chain fatty acids typical of the SP and mostly to lauric acid in BSFL. However, other oil active ingredients as antimicrobial peptides may converge with fatty acids in fighting against bacteria, especially Gram-positive ones, that appeared more sensitive.

Further research is needed to fully elucidate the role of the different natural products contained in the oil and to test how to potentiate their activity without adding chemical solvents as DMF or DMSO. According to the fact that insects have always been part of animal diets, their oils appear to be very good candidates to be used as feed additives/preservatives in animal husbandry.

## Figures and Tables

**Table 1:** Lipids contained in the larval samples.

Larval Samples	Dry Matter (DM) (%)	Rehydrated Weight (g) *	Lipids (g/100g DM)
Dried at 70 °C until constant weight	98.03	-	30.61
After water extraction at 70 °C	42.71	112	26.59
After water extraction at 100 °C	25.14	183	24.10

* weight of dried larvae 50 g.

**Table 2:** Lipids contained in BSFL samples after extraction with the Naviglio Extractor.

Naviglio Test Number	Weight of Sample before Extraction (g of DM)	Weight of Sample after Extraction (g of DM)	Extracted Lipids (g)	Extracted Lipids (% of Initial DM)
1	50	39	0.36	0.72
2	50	40	0.27	0.54
3	50	40	2.65	5.31
4	50	40	0.14	0.28

**Table 3:** Content of fatty acids in SP.

Fatty Acids	Notation	A	B	C	D
		M ± Ue	M ± Ue	M ± Ue	M ± Ue
Lauric acid	C12:0	0.06 ± 0.01	0.05 ± 0.01	0.06 ± 0.01	0.04 ± 0.01
Myristic acid	C14:0	0.18 ± 0.01	0.15 ± 0.01	0.14 ± 0.01	0.16 ± 0.01
Myristoleic acid	C14:1	0.03 ± 0.01	0.02 ± 0.01	0.03 ± 0.01	0.00 ± 0.00
Pentadecyclic acid	C15:0	0.07 ± 0.02	0.03 ± 0.01	0.03 ± 0.01	0.04 ± 0.01
Pentadecanoic acid	C15:1	0.07 ± 0.02	0.08 ± 0.02	0.07 ± 0.02	0.08 ± 0.02
Palmitic acid	C16:0	24.45 ± 2.38	23.34 ± 2.38	21.76 ± 2.38	22.07 ± 2.38
Palmitoleic acid	C16:1	1.56 ± 0.32	1.39 ± 0.32	1.14 ± 0.32	1.14 ± 0.32
Margaric acid	C17:0	0.16 ± 0.01	0.14 ± 0.01	0.17 ± 0.01	0.13 ± 0.01
Heptadecenoic acid	C17:1	0.04 ± 0.02	0.03 ± 0.02	0.03 ± 0.02	0.03 ± 0.02
Stearic acid	C18:0	4.35 ± 0.15	4.47 ± 0.15	5.01 ± 0.15	4.21 ± 0.15
Oleic acid	C18:1	31.17 ± 2.44	31.75 ± 2.44	32.40 ± 2.44	25.80 ± 2.44
Linoleic acid	C18:2(n-6)	4.64 ± 0.29	5.11 ± 0.29	6.29 ± 0.29	7.50 ± 0.29
Arachidic acid	C20:0	0.16 ± 0.02	0.17 ± 0.02	0.19 ± 0.02	0.19 ± 0.02
Eicosenoic acid	C20:1	0.02 ± 0.01	0.02 ± 0.01	0.04 ± 0.01	0.04 ± 0.01
Linolenic acid	C18:3(n-3)	32.68 ± 0.26	32.88 ± 0.26	32.25 ± 0.26	38.18 ± 0.26
Behenic acid	C22:0	0.01 ± 0.02	0.01 ± 0.02	0.01 ± 0.02	0.01 ± 0.02
Erucic acid	C22:1	0.08 ± 0.01	0.08 ± 0.08	0.11 ± 0.02	0.12 ± 0.02
Lignoceric acid	C24:0	0.03 ± 0.08	0.02 ± 0.01	0.05 ± 0.08	0.02 ± 0.08
Oleic acid, trans isomer	C18:1t	0.04 ± 0.06	0.03 ± 0.06	0.04 ± 0.06	0.03 ± 0.06
Linoleic acids, trans isomer	C18:2t	0.03 ± 0.06	0.07 ± 0.01	<0.01	<0.01
Linolenic acids, trans isomer	C18:3t	0.25 ± 0.10	0.22 ± 0.24	0.20 ± 0.10	0.24 ± 0.10
SFA/UFA ratio		0.42	0.40	0.38	0.37
ω6/ω3 ratio		0.14	0.16	0.19	0.20

Results represent the average value of two independent measurements analyses, as % area, (after repeatability check) ± uncertainty of measurement (Ue). A = 3–4 weeks old pupae (freshly produced pupae); B = 3–4 months old pupae; C = one year and three months old pupae; D = pupae residuals from reeling process; M = mean; Ue = expanded uncertainty associated with a test result based on a coverage factor K = 2 and a level of confidence of 95%.

**Table 4:** Chemical composition of *B. mori* pupae.

Parameters	Unit	A	B	C	D
Moisture and volatile matter	% m/m	5.89 ± 0.51	9.78 ± 0.26	7.73 ± 0.22	7.84 ± 0.31
Oil content	% m/m	30.01± 0.75	28.64 ± 0.75	24.37 ± 0.75	25.78 ± 0.75
Acidity	% oleic acid	2.4 ± 0.1	3.2 ±0.1	26.0 ± 1.2	2.8 ± 0.1
PV	mEq O_2_/kg	2.7 ± 0.4	6.1 ± 0.9	2.9 ± 0.4	13.3 ± 2.1

Results represent the average value of two independent measurements analyses (after repeatability check) ± uncertainty of measurement (Ue). PV = Peroxide Value, as determined on cold extracted oil; Ue = expanded uncertainty associated with a test result based on a coverage factor K =2 and a level of confidence of 95%.

**Table 5:** The inhibition growth-halo measurements (mm) obtained with agar diffusion test.

Treatments	*H. illucens*		*B. mori*	
	*B. subtilis*	*S. aureus*	*B. subtilis*	*S. aureus*
Oil/DMSO (1:1)	18.60 ± 1.04 A, B	12.60 ± 1.80	13.75 ± 1.89	13.30 ± 1.12A
OIL/DMF (1:1)	12.67 ± 0.12	9.60 ± 2.75	10.83 ± 2.39	7.30 ± 2.00

Means in the same column followed by the letter A were significantly different among groups (*p* < 0.05). The mean in the same row followed by the letter B was significantly different from the corresponding group (*p* < 0.05). Statistical analyses were performed by Mann-Whitney non-parametric test. Means and standard deviations are shown.

## Data Availability

The data presented in this study are available on request from the corresponding author. The data are not publicly available because they can be used by the Operational Group “Serinnovation” for future economic exploitation.

## Supplementary Materials

The following are available online at https://www.mdpi.com/2076-2615/11/3/783/s1, Figure S1: BSFL meal sedimentation, Figure S2: BSF whole larvae sedimentation 70 °C, Figure S3: BSF whole larvae sedimentation 100 °C, Figure S4: Mechanical pressing of BSFL and SP, Table S1: Fatty acids contained in BSF non-defatted meal and oil, Figure S5: Fatty acid (SFA, MUFA, PUFA) composition of silkworm oil and BSFL oil compared to available literature.
