# Supplementary material for: Mechanical Processing of Hermetia illucens Larvae and Bombyx mori Pupae Produces Oils with Antimicrobial Activity
Source: Animals (Basel). 2021 Mar 11;11(3):783. doi: 10.3390/ani11030783 (PMC8001418; doi:10.3390/ani11030783)
Supplement: Supplementary file 1 [file animals-11-00783-s001.pdf]

*animals*

MDPI

*Supplementary Material*

# Mechanical Processing of *Hermetia illucens* Larvae and *Bombyx mori* Pupae Produces Oils with Antimicrobial Activity

**Alessio Saviane [1], Luca Tassoni [2], Daniele Naviglio [3], Daniela Lupi [4], Sara Savoldelli [4], Giulia Bianchi [5], Giovanna Cortellino [5], Paolo Bondioli [6], Liliana Folegatti [7], Morena Casartelli [8], Viviana Teresa Orlandi [9], Gianluca Tettamanti [9] and Silvia Cappellozza [1,*]**

[1] Consiglio per la Ricerca in Agricoltura e l'Analisi dell'Economia Agraria, Centro di Ricerca Agricoltura e Ambiente (CREA-AA), 35143 Padova, Italy; alessio.saviane@crea.gov.it
[2] Istituto Zooprofilattico Sperimentale delle Venezie, 35020 Legnaro, Padova, Italy; ltassoni@izsvenezie.it
[3] Dipartimento di Scienze Chimiche, Università di Napoli "Federico II", 80126 Napoli, Italy; naviglio@unina.it
[4] Dipartimento Scienze per gli Alimenti, la Nutrizione e l'Ambiente, Università degli Studi di Milano, 20133 Milano, Italy; daniela.lupi@unimi.it (D.L.); sara.savoldelli@unimi.it (S.S.)
[5] Consiglio per la Ricerca in Agricoltura e l'Analisi dell'Economia Agraria, Centro di Ricerca Ingegneria e Trasformazioni Agroalimentari (CREA-IT), 20133 Milano, Italy; giulia.bianchi@crea.gov.it (G.B.); giovanna.cortellino@crea.gov.it (G.C.)
[6] Freelance Expert, 20133 Milano, Italy; paolo.bondioli1956@gmail.com
[7] Innovhub, Laboratorio sostanze grasse, derivati e tecnologie olearie, 20133 Milano, Italy; liliana.folegatti@mi.camcom.it
[8] Dipartimento di Bioscienze, Università degli Studi di Milano, 20133 Milano, Italy; morena.casartelli@unimi.it
[9] Dipartimento di Biotecnologie e Scienze della Vita, Università degli Studi dell'Insubria, 21100 Varese, Italy; viviana.orlandi@uninsubria.it (V.T.O.); gianluca.tettamanti@uninsubria.it (G.T.)
* Correspondence: silvia.cappellozza@crea.gov.it

**Citation:** Saviane, A.; Tassoni, L.; Naviglio, D.; Lupi, D.; Savoldelli, S.; Bianchi, G.; Cortellino, G.; Bondioli, P.; Folegatti, L.; Casartelli, M.; et al. Mechanical Processing of *Hermetia illucens* Larvae and *Bombyx mori* Pupae Allows to Obtain Oil with Antimicrobial Activity. *Animals* **2021**, *11*, 783. https://doi.org/10.3390/ani11030783

Academic editor: Laura Gasco

Received: 1 January 2021
Accepted: 4 March 2021
Published: 11 March 2021

**Publisher's Note:** MDPI stays neutral with regard to jurisdictional claims in published maps and institutional affiliations.

**Copyright:** © 2021 by the authors. Licensee MDPI, Basel, Switzerland. This article is an open access article distributed under the terms and conditions of the Creative Commons Attribution (CC BY) license (http://creativecommons.org/licenses/by/4.0/).

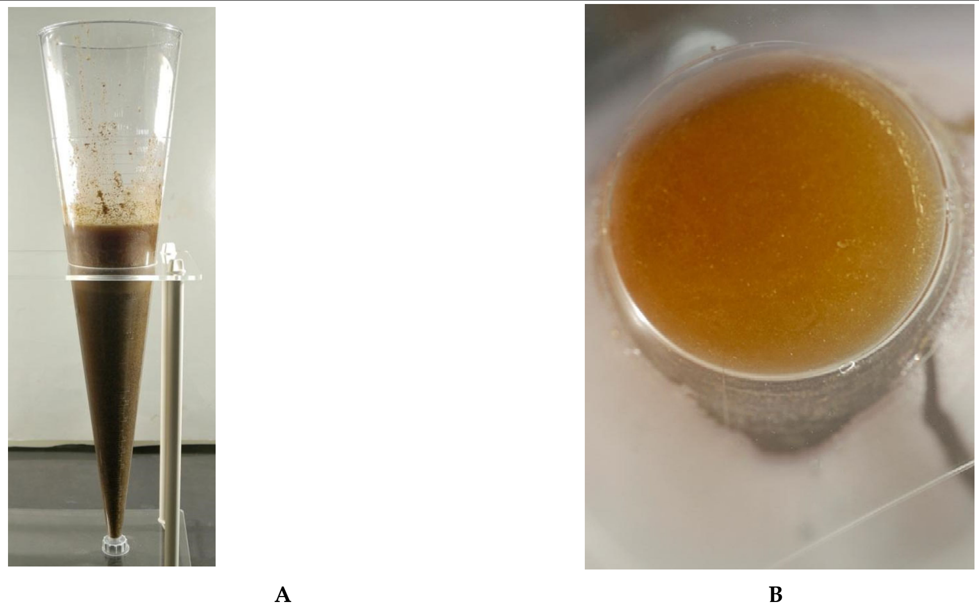


**Figure S1. A)** BSFL meal sedimentation; **B)** Surfacing of meal lipids.

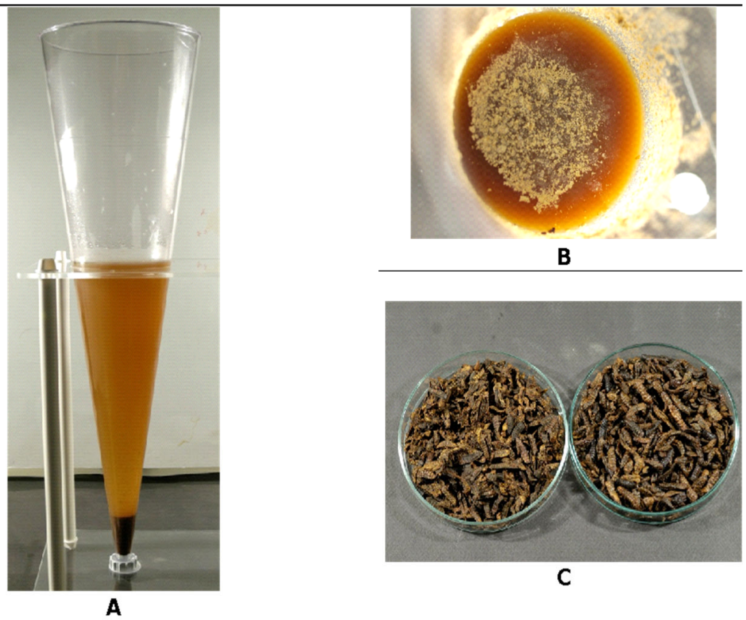


**Figure S2. A)** BSF whole larvae sedimentation; **B)** Surfacing of larvae lipids; **C**) Larvae before (left) and after (right) treatment

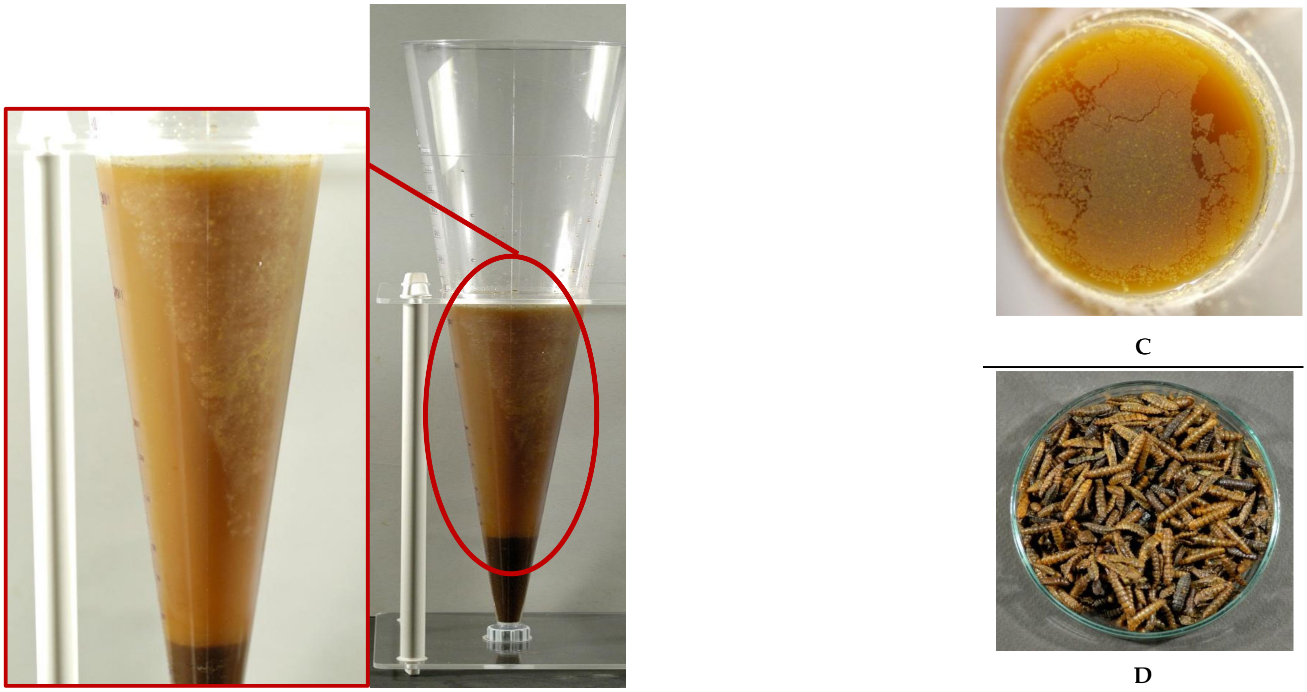


**A** **B**

**Figure S3. A–B)** BSF larvae sedimentation and solidified lipids distributed in almost all the water amount; **C)** Surfacing of larvae lipids; **D)** Larvae after 1 h extraction with water at 100 °C.

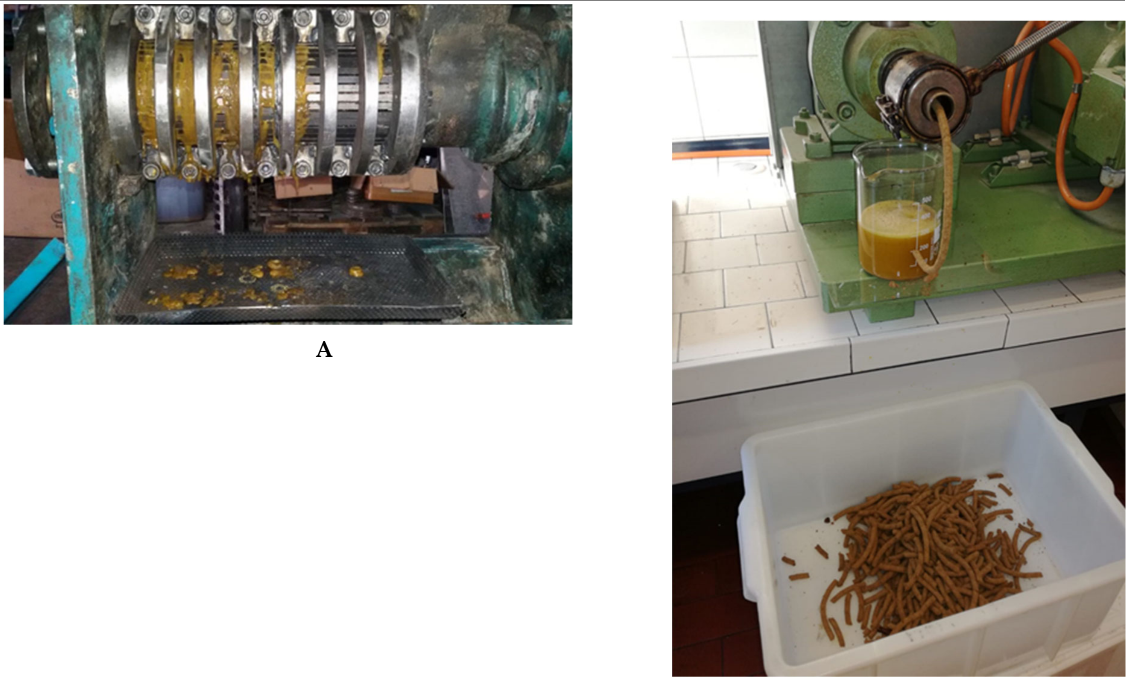


**B**

**Figure S4. A)** Mechanical pressing of *H. illucens* larvae; **B)** mechanical pressing of *B. mori* pupae.

**Table S1.** Fatty acids contained in BSF non-defatted meal and oil.

| Fatty Acids | Notation | Non-defatted Meal Mean ± SD | Extracted Oil Mean ± SD |
|---|---|---|---|
| Caproic | C6:0 | 0.02 ± 0.01 | 0.01 ± 0.00 |
| Enanthic | C7:0 | 0.02 ± 0.00 | 0.01 ± 0.00 |
| Caprylic | C8:0 | 0.03 ± 0.01 | 0.02 ± 0.00 |
| Pelargonic | C9:0 | 0.05 ± 0.01 | 0.04 ± 0.00 |
| Capric | C10:0 | 0.83 ± 0.00 | 0.89 ± 0.00 |
| Undecanoic | C11:0 | 0.03 ± 0.00 | 0.03 ± 0.00 |
| Undecenoic | C11:1 | 0.03 ± 0.00 | 0.03 ± 0.00 |
| Dodecanoic, anteiso | C12:0 anteiso | 0.03 ± 0.01 | 0.00 ± 0.00 |
| Lauric | C12:0 | 38.31 ± 0.00 | 42.45 ± 0.09 |
| Dodecenoic, cis | C12:1c | 0.06 ± 0.00 | 0.08 ± 0.00 |
| Dodecenoic, trans | C12:1t | 0.05 ± 0.00 | 0.06 ± 0.00 |
| Tridecanoic, iso | C13:0 iso | 0.03 ± 0.00 | 0.03 ± 0.00 |
| Tridecanoic, anteiso | C13:0 anteiso | 0.05 ± 0.00 | 0.05 ± 0.00 |
| Tridecanoic | C13:0 | 0.05 ± 0.00 | 0.04 ± 0.00 |
| Tetrodecanoic, iso | C14:0 iso | 0.08 ± 0.01 | 0.06 ± 0.00 |
| Myristic | C14:0 | 7.94 ± 0.00 | 8.23 ± 0.01 |
| Tetradecenoic, trans | C14:1t | 0.08 ± 0.00 | 0.07 ± 0.00 |
| Tetradecenoic, n-7 | C14:1n7 | 0.05 ± 0.02 | 0.04 ± 0.00 |
| Tetradecenoic, n-5 | C14:1n5 | 0.27 ± 0.00 | 0.30 ± 0.00 |
| Pentadecanoic, iso | C15:0 iso | 0.11 ± 0.01 | 0.08 ± 0.00 |
| Pentadecanoic, anteiso | C15:0 anteiso | 0.2 ± 0.01 | 0.15 ± 0.00 |
| Pentadecanoic | C15:0 | 0.33 ± 0.00 | 0.28 ± 0.00 |
| Pentadecenoic, trans | C15:1t | 0.12 ± 0.03 | 0.11 ± 0.01 |
| Esadecanoic iso | C16:0 iso | 0.12 ± 0.01 | 0.09 ± 0.00 |
| Palmitic | C16:0 | 16.93 ± 0.03 | 15.35 ± 0.03 |
| Hexadecenoic, n-9 | C16:1n9 | 0.37 ± 0.00 | 0.32 ± 0.00 |
| Hexadecenoic, n-7 | C16:1n7 | 4.17 ± 0.01 | 4.02 ± 0.01 |
| Hexadecenoic, trans | C16:1t | 0.02 ± 0.01 | 0.02 ± 0.00 |
| Heptadecanoic, iso | C17:0 iso | 0.09 ± 0.01 | 0.08 ± 0.00 |
| Heptadecanoic, anteiso | C17:0 anteiso | 0.13 ± 0.00 | 0.10 ± 0.00 |
| Heptadecanoic | C17:0 | 0.36 ± 0.01 | 0.32 ± 0.00 |
| Heptadecenoic, n-7 | C17:1n7 | 0.33 ± 0.03 | 0.28 ± 0.01 |
| Heptadecenoic, trans | C17:1t | 0.06 ± 0.01 | 0.05 ± 0.00 |
| Hexadecadienoic | C16:2 | 0.06 ± 0.00 | 0.06 ± 0.01 |
| Stearic | C18:0 | 3.31 ± 0.00 | 2.95 ± 0.01 |
| Oleic | C18:1n9 | 15.14 ± 0.02 | 13.53 ± 0.03 |
| Octadecenoic, n-7 | C18:1n7c | 0.83 ± 0.01 | 0.78 ± 0.01 |

| | | | |
|---|---|---|---|
| Linoleic | C18:2n6 linoleic | 6.02 ± 0.01 | 5.3 ± 0.00 |
| Nonadecanoic | C19:0 | 0.02 ± 0.00 | 0.02 ± 0.00 |
| a-Linolenic | C18:3n3 | 0.79 ± 0.00 | 0.8 ± 0.03 |
| Conjugated linoleic 9c, 11t | CLA c9t11 | 1.38 ± 0.01 | 1.51 ± 0.01 |
| Conjugated others | CLA | 0.03 ± 0.00 | 0.02 ± 0.01 |
| Conjugated linoleic trans, trans | CLAtt | 0.03 ± 0.00 | 0.04 ± 0.00 |
| Arachidic | C20:0 | 0.09 ± 0.01 | 0.09 ± 0.02 |
| Octadecatetraenoic | C18:4n4 | 0.3 ± 0.01 | 0.36 ± 0.00 |
| Eicosenoic | C20:1n9 | 0.07 ± 0.01 | 0.07 ± 0.00 |
| Eicosadenioic | C20:2n6 | 0.04 ± 0.01 | 0.04 ± 0.00 |
| Eicosatrienoic | C20:3n6 | 0.39 ± 0.00 | 0.63 ± 0.17 |
| Arachidonic | C20:4n6 | 0.07 ± 0.00 | 0.05 ± 0.01 |
| Eicosapentaenoic | C20:5n3 EPA | 0.04 ± 0.01 | 0.05 ± 0.01 |
| Behenic | C22:0 | 0.05 ± 0.02 | 0.04 ± 0.00 |
| SFA/UFA ratio | | 2.25 | 2.49 |
| ω6/ω3 ratio | | 5.76 | 4.95 |

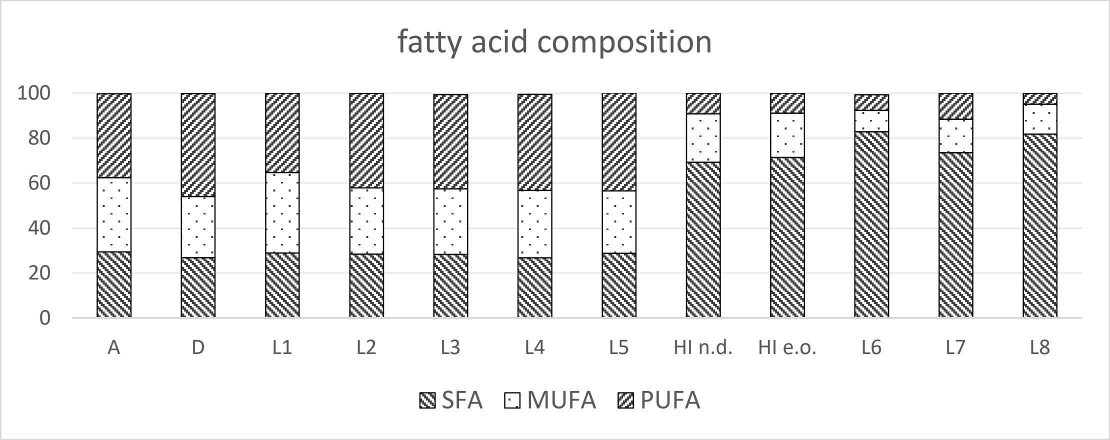


**Figure S5:** Fatty acid (SFA, MUFA, PUFA) composition of silkworm oil and BSFL oil compared to available literature. A = experimental thesis A (see Table 4); D = experimental thesis (see Table 4). L1 [50]; L2, L3 [51]; L4 [52]; L5 [53], Hi n.d. = *H. illucens* non-defatted meal (see Table 3); Hi e.o. = *H. illucens* extracted oil (see Table 3); L6 [19]; L7 [54]; L8 [55].
